# Effect of Vitamins D and E on the Proliferation, Viability, and Differentiation of Human Dental Pulp Stem Cells: An In Vitro Study

**DOI:** 10.1155/2020/8860840

**Published:** 2020-07-01

**Authors:** Lina M. Escobar, Zita Bendahan, Andrea Bayona, Jaime E. Castellanos, María-Clara González

**Affiliations:** ^1^Unidad de Manejo Integral de Malformaciones Craneofaciales (UMIMC), Facultad de Odontología, Universidad El Bosque, Bogotá, Colombia; ^2^Posgrado de Ortodoncia, Facultad de Odontología, Universidad El Bosque, Bogotá, Colombia; ^3^Vicerrectoría de Investigaciones, Universidad El Bosque, Bogotá, Colombia

## Abstract

**Introduction:**

The aim of the present study was to determine the effects of vitamins D and E on the proliferation, morphology, and differentiation of human dental pulp stem cells (hDPSCs).

**Methods:**

In this in vitro experimental study, hDPSCs were isolated, characterized, and treated with vitamins D and E, individually and in combination, utilizing different doses and treatment periods. Changes in morphology and cell proliferation were evaluated using light microscopy and the resazurin assay, respectively. Osteoblast differentiation was evaluated with alizarin red S staining and expression of RUNX2, Osterix, and Osteocalcin genes using real-time RT-PCR.

**Results:**

Compared with untreated cells, the number of cells significantly reduced following treatment with vitamin D (49%), vitamin E (35%), and vitamins D + E (61%) after 144 h. Compared with cell cultures treated with individual vitamins, cells treated with vitamins D + E demonstrated decreased cell confluence, with more extensive and flatter cytoplasm that initiated the formation of a significantly large number of calcified nodules after 7 days of treatment. After 14 days, treatment with vitamins D, E, and D + E increased the transcription of RUNX2, Osterix, and Osteocalcin genes.

**Conclusions:**

Vitamins D and E induced osteoblastic differentiation of hDPSCs, as evidenced by the decrease in cell proliferation, morphological changes, and the formation of calcified nodules, increasing the expression of differentiation genes. Concurrent treatment with vitamins D + E induces a synergistic effect in differentiation toward an osteoblastic lineage.

## 1. Introduction

Currently, in the field of orthodontics, the study of mechanisms accelerating dental movement is being highlighted to enable a shortened treatment time and thus reduce side effects resulting from long periods of orthodontic treatment, such as tooth decay, periodontal disease, and root resorption [1].

Several methods have been developed to accelerate orthodontic tooth movement, including surgeries, flaps, periodontal invasion, infections, and surgical site interventions, such as osteotomy and corticotomy. However, the use of less invasive techniques such as microperforations and piezocision, or the use of substances such as prostaglandin, hormones, opioids, and vitamins, has recently been explored. These substances are proposed to increase dental movement mainly through the induction of osteoclast formation; however, side effects such as toxicity, root resorption, and recurrence, as well as the mechanisms of action of these approaches, remain unclear [2].

For orthodontists, it would be useful to generate rapid movement under ideal conditions allowing optimal results in the shortest possible time. However, it is also crucial that balanced bone remodeling occurs, leading to appropriate bone resorption induced by osteoclastogenesis with appropriate osteoblast-mediated bone formation, in both the pressure and tension zones, to ensure the stability and complete reconstruction of placental tissues.

Vitamin D is a potent stimulator of both apposition and bone resorption. During bone apposition, vitamin D demonstrates immunomodulatory actions by stimulating osteoblasts that generate bone mineralization. Regarding bone resorption, vitamin D, in the presence of the parathyroid hormone (PTH), is shown to stimulate differentiation and increase osteoclast activity. Therefore, vitamin D induces osteoclasts to produce bone resorption that facilitates dental movement, as well as bone formation that allows tooth stabilization. However, the biological mechanism that induces osteoblast and osteoclast activities mediated by vitamin D remains elusive [3].

Vitamin E is a fat-soluble antioxidant molecule that inhibits lipid peroxidation by capturing reactive oxygen species, believed to possess protective abilities against arteriosclerotic changes and aging processes. Tocopherol is the predominant isoform of vitamin E and a natural agent with antioxidant and anti-inflammatory properties, proposed to act as a stimulating factor for osteoblast proliferation and maturation [4]. However, these results are contradictory, as other studies indicate that *α*-tocopherol and *δ*-tocopherol inhibit osteoblast differentiation, especially from early stages to the osteoid production stage [5].

Human dental pulp stem cells (hDPSCs) were chosen for this study since they constitute an attractive source of multipotent mesenchymal stem cells owing to their high proliferation rate and multilineage differentiation potential. They are mesenchymal stem cells (MSCs) exhibiting similar features to bone marrow-derived mesenchymal stem cells (BMMSCs), including clonogenicity and capability of self-renewal, although they have greater proliferation capacity and ease obtaining than BMMSCs [6]. Additionally, they have a multilineage differentiation potential making them emerging sources of multipotent cells when compared to BMMSCs. DPSCs possess high potential to differentiate into various cells including neuronal cells, chondroblasts, adipocytes, and osteoblasts, suggesting that they are ideal for tissue engineering, osteogenesis, and regenerative medicine [7].

In orthodontics, several investigations have focused on the study of vitamin D for the acceleration of dental movement. However, evidence on the effect of vitamin D and vitamin E on the differentiation of mesenchymal cells in vitro is lacking. Furthermore, it remains to be elucidated whether these vitamins produce an agonistic or antagonistic effect on the viability, proliferation, and differentiation of mesenchymal cells toward an osteoblastic lineage.

Hence, the aim of this study was to determine whether the stimulus of vitamin D and vitamin E alters the proliferation, viability, and differentiation of hDPSCs toward an osteoblast phenotype.

## 2. Materials and Methods

### 2.1. Culture of Mesenchymal Cells from Dental Pulp

Dental pulp was obtained from sound premolars of three individuals aged between 18 and 20 years, with prior informed consent, who underwent tooth extraction for orthodontic treatment. Cell isolation was performed according to the protocol of Gronthos et al. [8]. Briefly, the teeth were decontaminated by immersion in 5% sodium hypochlorite and sectioned with a handpiece to recover the dental pulp. The obtained explants were placed in HyClone™ Dulbecco's Low Glucose Modified Eagles Medium (DMEM; Thermo Fisher Scientific), supplemented with fetal bovine serum (FBS) (Hyclone, Thermo Fisher Scientific) and antibiotics. A dissociation medium containing collagenase (3 mg/mL) (Sigma-Aldrich, USA) and dispase (4 mg/mL) (Gibco) was used for 16 h, in a humidified atmosphere with 5% CO_2_ at 37°C. Subsequently, the cell suspension was centrifuged, and the pellet was resuspended in medium to be seeded in 25 cm^2^ flasks until 80% confluence was achieved [8].

### 2.2. Phenotypic and Functional Characterization of Mesenchymal Cells

The characterization of the mesenchymal cells was performed according to the following criteria.

  Morphological characterization: mesenchymal cells must be elongated, spindle-shaped, narrow, and fibroblast-like, with a central nucleus and abundant lysosomes [9].
  Characterization by flow cytometry: several membrane markers were used and detected by flow cytometry. Previously, cultured cells were trypsinized, centrifuged, and resuspended in 100 *µ*L of a saline buffer. Subsequently, the cells were incubated with 10 *µ*L of fluorochrome-coupled monoclonal antibodies for CD34, CD45, CD73, CD90, and CD105 and mouse isotype control antibodies (FITC-IgG1, PE-IgG1, APC-IgG1 PerCP, IgG1, and PerCP-IgG2a) from the phenotyping kit for mesenchymal stem cells (MSCs; Miltenyi Biotec, Bergisch Gladbach, Germany). Samples were processed using a FACS Calibur flow cytometer (BD Biosciences, San Jose, CA, USA). A homogeneous population was characterized by presenting cells positive for CD73, CD90, and CD105 and negative for CD34 and CD45 [10].

### 2.3. Mesenchymal Stem Cell Treatment with Vitamins E and D

hDPSCs were cultured in 25 cm^2^ culture flasks until 80% confluence was achieved, and subsequently dissociated with 0.25% trypsin (DIFCO) and seeded in 12-well microplates, with 5000 cells per well. These cells were treated with different concentrations of vitamin D (1 × 10^−7^, 1 × 10^−8^, and 1 × 10^−9^M Sigma) and vitamin E (12, 6, 3, 1.5, and 0.75 *µ*M *a*-tocopherol Sigma) for 48, 72, 96, 120, and 144 h. Experiments were performed in triplicate and repeated twice.

### 2.4. Determination of Viability and Proliferation of MSCs

The live and dead cells were counted with a hemocytometer using the trypan blue dye exclusion technique. The number of cells, as well as cell proliferation, was determined using the resazurin fluorometric test. The resazurin solution (4.4 *μ*g per well) was added to control and treated cells and incubated at 37°C for 4 h; subsequently, they were analyzed using a Tecan Infinite M2000 Pro reader at 535–595 nm excitation-emission wavelengths [11].

### 2.5. Differentiation and Characterization of Osteoblastic Cells

#### 2.5.1. Osteoblastic Cell Differentiation Induced by Differentiation Medium (DM)

The differentiation control group was grown in osteogenic induction medium containing DMEM supplemented with 10% FBS, 100 *µ*g/mL penicillin, 100 *µ*g/mL streptomycin, 0.1 *µ*M dexamethasone (Sigma-Aldrich), 5 mM *β*-glycerophosphate (Santa Cruz, CA, USA), and 50 *µ*g/mL ascorbic acid (Sigma-Aldrich) with no addition of vitamin D or E. These cells were treated with the differentiation medium (positive control of differentiation) for 7, 14, and 21 days in a humidified atmosphere with 5% CO_2_ at 37°C. Negative control cells were maintained in a culture medium lacking osteoblastic differentiation factors [12].

#### 2.5.2. Evaluation of Osteoblast Gene Expression by Quantitative RT-PCR

Following treatment of MSCs with vitamins D, E, and D + E, the Quick-RNA MicroPrep kit (Zymo Research USA) was used for the extraction of total RNA at 7, 14, and 21 days, according to manufacturer's protocol. Subsequently, cDNA was obtained by reverse transcription using the ProtoScript ll First Strand cDNA Synthesis cDNA kit protocol (BIOHAUS S.A.S). To determine the expression levels of runt-related transcription factor 2 (RUNX2), Osterix (OSX), and Osteocalcin (OCN), RT-PCR was performed with the Luna Universal real-time RT-PCR master mix system (New England Biolabs, USA) using the CFX96 Real-Time Thermal Cycler detection system (Bio-Rad; Hercules, CA, USA). The amplification conditions were 3 min at 95°C and 50 cycles of 10 s at 95°C, 30 s at 60°C, and 20 s at 72°C, and finally, 5 s at 65°C, and 5 s at 95°C. The primers used are presented in Table 1. PCR efficiencies were calculated using LinRegPCR (Academic Medical Center, Amsterdam, The Netherlands), and relative quantification of expression was performed following the Scheffe method [13].

#### 2.5.3. Evluation of Mineralization In Vitro

Each group of cells was fixed with paraformaldehyde (PFA) and stained with 2% alizarin red S based on the protocol established by Gregory et al. [14]. Subsequently, the dye was extracted by washing with phosphate-buffered saline once. The formation of calcified nodules was analyzed using an inverted microscope, and the extracted dye absorbance was quantified using the Infinite M200 multiplate reader Infinite M200, Tecan (Männedorf, Suiza) at 550 nm [14].

### 2.6. Statistical Analysis

Data are expressed as the mean ± SD. A value of *p* < 0.05 was considered significant between the experimental groups. To analyze differences between the various groups with normal distribution, Student's *t*-test and ANOVA were used. For groups demonstrating nonnormal distribution, the Mann-Whitney *U* test was performed. All experiments were carried out using two independent cultures, with three replicates per condition. Statistical analysis was performed using the SPSS software, version 21.0 (SPSS, Chicago, IL, USA).

## 3. Results

### 3.1. Vitamins D and E Decreased hDPSC Proliferation Proportional to the Dose and Time of Treatment

**Figure 1:**
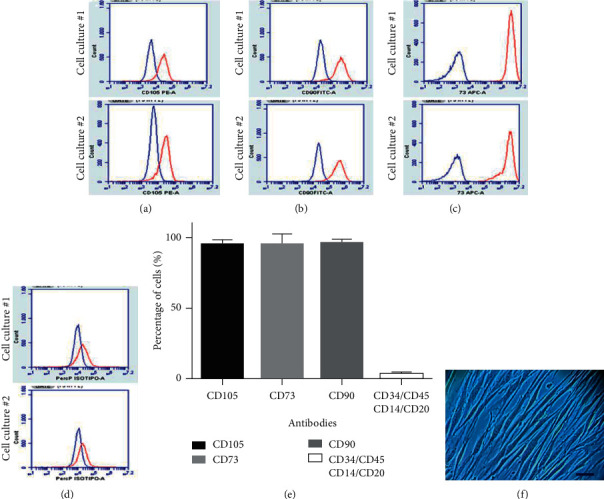
hDPSCs characterization. Flow cytometric histograms of two hDPSC primary cultures with positive surface markers for (a) CD105, (b) CD90, and (c) CD73 and (d) negative for CD34, CD14, CD20, and CD45. (e) Percentage of positive cells for each antibody. 95% of the cells were positive for CD73 and CD90, and less than 2% were positive for CD34, CD45, CD14, and CD20. (f) Photomicrograph of hDPSCs with fibroblast phenotype, 7 days after culture. Values are presented in averages and standard deviations (SD), from two independent experiments. Bar 200 *μ*m.

hDPSCs were characterized, and the mesenchymal stem cell marker profile (CD73+, CD90+, CD105+, CD14−, CD20−, CD34−, CD45−) (Figures 1(a)–1(e)) and elongated fibroblast-like morphology were determined (Figure 1(f)).

Initially, the effect of treatment with vitamin D or vitamin E administered individually, on the proliferation of hDPSC at different times, was determined.

**Figure 2:**
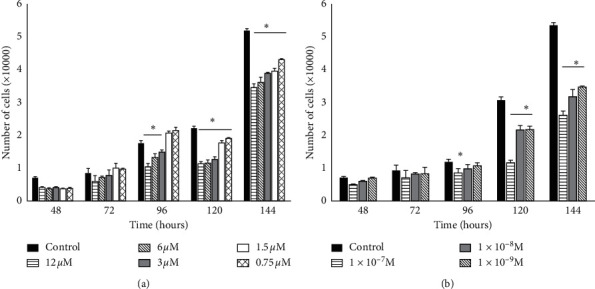
Reduction in the number of hDPSCs induced by treatment with different doses of vitamins D and E (a). Vitamin E produced a decrease in the number of cells directly proportional to the concentration used. Significant differences were observed from 96 h at doses of 12, 6, and 3 *μ*M compared with the control group. (b) Vitamin D treatment reduced the number of hDPSCs in all doses used after 120 h in comparison with the control group. Asterisks show significant differences (*p* < 0.05). Data are is expressed as averages ± DSSD.

hDPSC treatment with vitamin E significantly reduced cell proliferation at all doses after 120 h of treatment, with a maximal decline of 31% observed with the highest concentration (12 *µ*M) (Figure 2(a)). Conversely, vitamin D, at a dose of 1 × 10^−7^ M, induced significantly lower cell proliferation after 96 h. After 120 h, all treatment concentrations induced a significant decrease in cell number, with the greatest decline in cell proliferation (53%) observed in cells treated with vitamin D at 1 × 10^−7^ M when compared to proliferation in the untreated group (control) (Figure 2(b)). No evident cell death was observed in the experimental groups treated with vitamin D or E at different treatment times.

### 3.2. Simultaneous Treatment with Vitamins D and E Induced a Greater Decrease in Cell Proliferation than that Observed with Individual Applications

Given that 1 × 10^−7^ M of vitamin D and 12 *µ*M of vitamin E induced the greatest changes in proliferation and morphology of hDPSCs during the different times, these doses were chosen to be used in the simultaneous treatment with the two vitamins.

**Figure 3:**
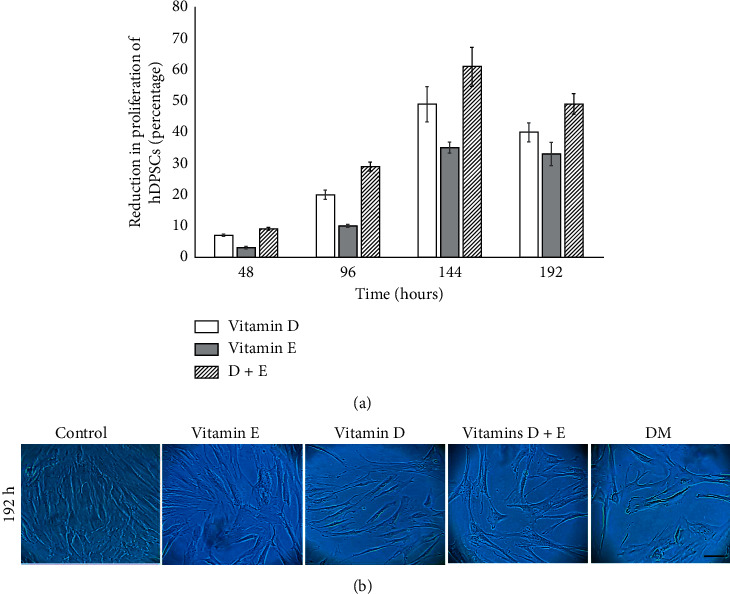
Changes in hDPSC proliferation and morphology induced by treatment with vitamins D and E. (a) Percentage reduction in proliferation induced by treatment with vitamin D (10^−7^M), vitamin E (12 *μ*M), and the two vitamins simultaneously (D + E) during 192 h of treatment. Greater reduction in the proliferation of cells treated with vitamins D + E (61% at 144 h and 49% at 192 h) can be observed compared to that observed when applying vitamin D (49% at 144 h and 40% at 192 h) and vitamin E (35% at 144 h and 33% at 192 h) individually. All treatment groups showed statistically significant differences between them and with the control from 96 h (*p* < 0.05). (b) Microphotograph of hDPSC treated with vitamin D, vitamin E, and vitamins D + E and differentiation medium (DM) during 192 h. Cells treated with vitamins D and E simultaneously (D + E) presented less cell confluence with a larger and flatter cytoplasm. Bar 200 *μ*m.

Treatment with vitamin D (1 × 10^−7^ M), vitamin E (12 *µ*M), and vitamins D + E decreased proliferation by 49%, 35%, and 61%, respectively, after 144 h, and by 40%, 33%, and 49%, respectively, after 192 h of treatment, with no evident cell death observed in the experimental groups. Simultaneous administration of vitamins D + E significantly reduced the number of cells (*p* < 0.05) in the control group, as well as groups individually treated with vitamins D and E (Figure 3(a)).

Cell cultures concurrently treated with vitamins D and E (D + E) demonstrated lower cell confluence, with a larger and flatter cytoplasm. In contrast, cells treated with vitamin D presented a more elongated morphology (Figure 3(b)).

### 3.3. Treatment with Vitamins D and E Individually and Concurrently Led to Osteoblastic Differentiation of hDPSCs

**Figure 4:**
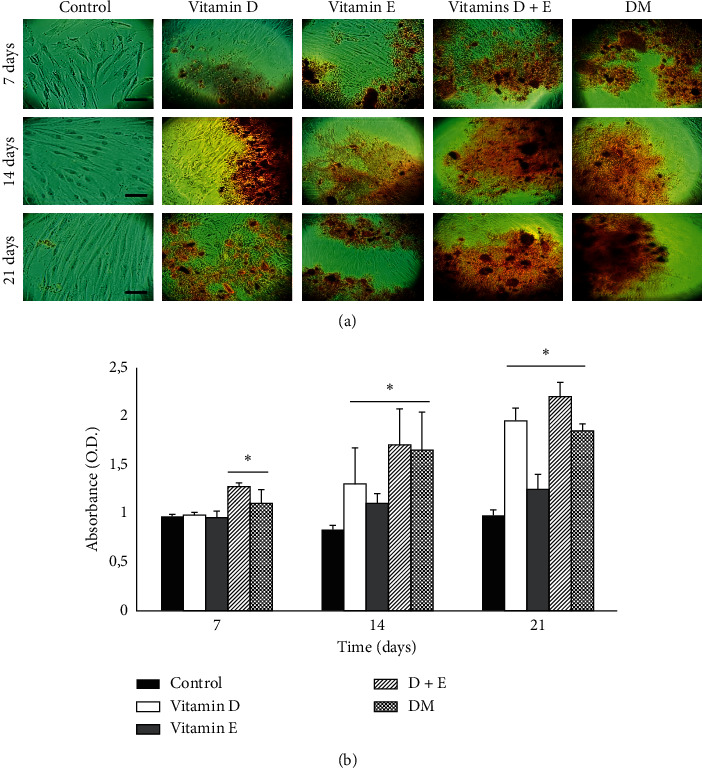
Mineralization of the extracellular matrix. (a) Mineralization was determined by alizarin red S staining. Strong matrix staining was observed in photomicrographs, which indicated the apparent formation of calcification nodules. (b) Measurement of absorbance (Optical Density, OD) of alizarin red stain extracted from cells under different treatments at 7, 14, and 21 days. Differentiation medium (DM). Asterisks show significant differences (*p* < 0.05) with respect to the control group. Data are expressed as averages ± SD. Bar 200 *μ*m.

To evaluate the osteoblastic activity, alizarin red S staining determined the formation of calcified nodules in cells treated with vitamins D, E, and D + E for 7, 14, and 21 days (Figure 4). After 7 days, the initial formation of calcified nodules in cells treated with vitamins D + E was greater than that observed in the cells individually treated with the vitamins (Figure 4(a), 7 days). At 14 days, a significant increase in calcified nodule formation was associated with vitamin D treatment when compared with that observed at 7 days (Figure 4(a), 14 days).

Moreover, in all experimental groups, an increase in the number of calcified nodules was observed at 21 days (Figure 4(a), 21 days). On quantifying the absorbance of alizarin red S extracted from the cells, significant differences were observed after 7 days between cells treated with vitamins D + E and the DM when compared with the control group. At 14 and 21 days, a significant increase in the amount of Alizarin red S was observed in all experimental groups relative to the control group (Figure 4(b)).

**Figure 5:**
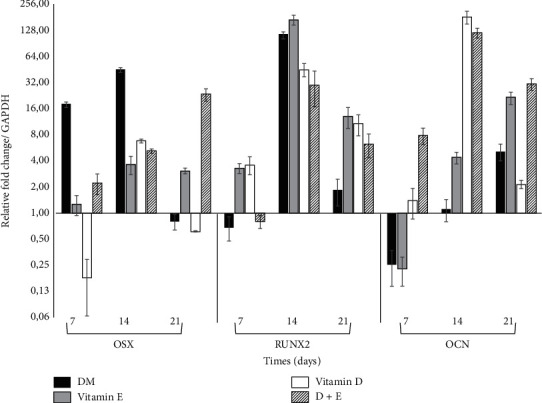
Relative gene expression quantitation during differentiation: quantification of the relative expression of specific osteoblasts and mineralizing transcripts runt-related transcription factor 2 (*RUNX*2), Osterix (*OSX*), and Osteocalcin (*OCN*) was performed in hDPSCs treated with vitamins D, E, and D + E during 7, 14, and 21 days. Data is expressed in relation to the expression levels of the glyceraldehyde-3-phosphate dehydrogenase (*GAPDH*) gene and as a positive control, and cells treated with differentiation medium (DM) were analyzed.

 To understand the role of vitamins D and E in osteoblast differentiation, we compared their effects on osteoblastic marker expression using real-time PCR analysis. Following 14 days of treatment, increased expression of the OSX gene was observed in all experimental groups; later, decreased expression was evident, except in the group concurrently treated with the two vitamins (vitamins D + E), in which the OSX expression continued increasing (Figure 5). Conversely, after 14 days, the expression of RUNX2 increased significantly in all experimental groups. Furthermore, vitamin E treatment produced the greatest increase observed at 14 days (164 times). At 21 days, the expression of RUNX2 decreased in all groups (Figure 5). Finally, the OCN gene demonstrated increased expression in all experimental groups at 14 days. Subsequently, OCN expression decreased after 21 days in the vitamin D group, as well as the group concurrently treated with the two vitamins (D + E). After 21 days of treatment, cell groups treated with the DM and vitamin E demonstrated an increase in OCN gene expression (Figure 5).

## 4. Discussion

Orthodontic-mediated tooth movement is dependent on the remodeling of tissues surrounding the teeth following the application of mechanical forces. For dental movement to occur, the application of mechanical forces capable of activating bone and related cells generating inflammatory changes in periodontal tissue is crucial, triggering the desired dental movement. Currently, methods are being investigated to accelerate the movement, utilizing some invasive methods such as osteotomies, corticotomies, and others, such as piezoincisions and micro-osteoperforations. Additionally, mechanical methods such as vibration and low-frequency lasers and chemicals, including the application of prostaglandins, hormones, opioids, and vitamins, have been explored [15].

Vitamin D is one of the vitamins investigated to accelerate dental movement and is a powerful regulator of bone development and metabolism, participating in calcium homeostasis and significantly increasing bone formation in the reabsorbed area of alveolar bone after tooth movement. Following orthodontic treatment, vitamin D can promote the restoration of the supporting teeth tissue [16]. Additionally, it has been determined that vitamin D, in its active form, plays a particularly important role in the bone tissue as it can stimulate both bone formation and reabsorption, regulating bone turnover and acting on both osteoblasts and osteoclasts [17]. Notably, the interaction between vitamin D and osteoblasts is complex. The presence of the vitamin D receptor in osteoblasts allows direct effects, but these effects vary with the dose and time of treatment and the osteoblast origin. The effect of vitamin D on differentiation and mineralization is primarily stimulatory in human and rat osteoblasts and inhibitory in murine osteoblasts. Although most research indicates an inducing effect of osteoclasts and osteoblasts, some studies have suggested that the main in vivo pharmacological action of vitamin D, at least in a high state of bone turnover, such as estrogen deficiency, is to suppress osteoclastic bone resorption [18]. This induces uncertainty regarding the role vitamin D plays in bone resorption, or deposition and mineralization around the tooth undergoing orthodontic movement. In the present study, hDPSCs treated with vitamin D demonstrated a significant dose-dependent decrease in proliferation. The decrease in the number of cells was significant with the vitamin D 1 × 10^−7^ M treatment from 96 h; at 120 h, all treatment concentrations (1×10^−5^M, 1 × 10^−6^ M, and 1 × 10^−7^ M) induced a significant decline in cell number. This reduced cell number is associated with a decline in cell proliferation as no evident cell death was observed in any experimental group. Similarly, Atkins et al. have demonstrated that 1*α*-25-dihydroxyvitamin D3 (1,25-D3), an important metabolite of vitamin D, considerably reduces the cell proliferation rate at a concentration of 1 × 10^−3^ M, indicating that this is an important physiological regulator of human osteoblast proliferation [19]. Ji et al. have employed in vitro experiments to clarify how 1,25-D3 induces osteogenic differentiation in stem cells from the human periodontal ligament, noting a decrease in cell proliferation when compared to the control group at different concentrations (0.01, 1, and 10 nM) following 24, 48, and 72 h [3]. Wang et al. have investigated the effect of 1,25-D3 (0.01 and 0.02 nmol/L) on osteoblasts for 7 to 14 days and reported a significant increase in the viability of osteoblasts; however, depending on the dose administered, the results varied and failed to produce significant cell differentiation [20]. Unlike previous findings, another study using osteoblast-like MC3T3-E1 cells treated with concentrations of vitamin D 1 × 10^−10^, 1 × 10^−12^, and 1 × 10^−14^ M failed to inhibit cell growth, demonstrating a proliferation rate greater than that observed in the control group at all concentrations. However, an increase in the expression of markers associated with cell differentiation was observed [21]. Therefore, the effects of vitamin D directly depend on the doses administered, inducing the osteogenic differentiation of stem cells; however, depending on the medium, the concentration and type of cells may or may not produce a considerable decrease in cell proliferation.

Regarding the in vitro effect of vitamin E on MSC proliferation, we observed a significant decline in the number of cells at all doses (from 0.75 to 12 *µ*M), from 120 h of treatment. Studies evaluating changes in cell proliferation induced by vitamin E treatment are contradictory: Fujita et al. have investigated the action of vitamin E in mice deficient in the transfer protein of *α*-tocopherol in comparison with a wild type mouse model, documenting that osteoblastic differentiation and proliferation were not altered by vitamin E [22]. Additionally, Urban et al. have observed that vitamin E treatment did not increase the osteoblast proliferation in vitro; however, they argue that high doses of this vitamin can generate toxic effects preventing osteoblast differentiation and proliferation [23]. Conversely, Ahn et al. have reported that the proliferation of human MSCs increases after incubation with *α*-tocopherol [24]. In a study by Wan Hasan et al., preosteoblastic MC3T3-E1 cells treated with annatto-derived tocotrienol (a vitamin E derivative), at doses of 5, 10, and 20 *µ*g/mL for 3 to 6 days, demonstrated a significant decrease in cell proliferation and cell viability [4]. In our study, vitamin E treatment failed to reduce the number of living cells at any dose evaluated.

Furthermore, morphological changes were evaluated in cells treated with vitamins D and E. A lower cell density was observed, directly proportional to the concentration used, and cells presented greater extensions, with polygonal and abundant cytoplasm. These changes are similar to those reported by Wan Hasan et al., who detected morphological changes in MC3T3-E1 preosteoblastic cells treated with a vitamin E derivative (0.001–1 *µ*g/mL), forming larger and more cuboidal cells when compared to control cells [4, 25]. Additionally, Khanna-Jain et al. have determined that, at the morphological level, vitamin D induces the proliferation of pulp and dental follicular cells with a triangular, stellate, or spindle-shaped morphology, in approximately 1-2 weeks, subsequently presenting a network arrangement [26]. Meanwhile, Lou et al. have investigated the hypothesis that 1*α*, 25-dihydroxyvitamin D3 plays a fundamental role in mesenchymal cell morphology; cells cultured at a lower density in normal growth medium appeared larger, flatter, and spindle-shaped, while at a higher density following a 21-day culture, they presented a fibroblast-like morphology, depending on the dose administered. For example, 500 nM induces significant changes after 6 days, acquiring a more polygonal shape, a characteristic associated with osteoblastic cells [25].

Currently, no studies have evaluated the combined action of vitamin D and vitamin E on mesenchymal cell differentiation. The present study describes, for the first time, the changes induced utilizing this combination. We observed that vitamins D + E treatment induced a decrease in cell proliferation by 49% after 8 days of treatment (192 h), a value greater than that observed with individual vitamin treatment.

Regarding the effect of these vitamins on osteoblastic differentiation and mineralization in vitro, more mineralized nodule formation was observed with alizarin red S staining after combined vitamin treatment when compared with individual treatments and the control.

After 14 days of treatment, vitamin D-treated cells demonstrated a significant increase in the formation of calcified nodules. Similarly, De Kok et al. have determined that this vitamin induces osteoblastic differentiation in human MSCs cultured for a period of 14 to 21 days, with a greater formation of mineralized nodules after this period [27]. Additionally, Khanna-Jain et al. have reported that pulp and follicle stem cells form a mineralized matrix when treated with vitamin D3 metabolites [26]. In another study, treatment with vitamin D has demonstrated an increase in the mineralized nodule number [28]. Ji et al. have shown an increase in the number of mineralized nodules in human periodontal ligament mesenchymal cells treated with vitamin D [3]. Similarly, we observed that vitamin E increased the number of calcified nodules similar to that induced by vitamin D treatment. Furthermore, Wan Hasan et al. have demonstrated similar results in MC3T3-E1 cells treated with different concentrations of vitamin E (0.001–1 *µ*g/mL), where the intensity of positive alizarin red S staining increased from day 3 to day 21 in all experimental groups [4].

Vitamin D induced an increase in the expression of cell differentiation genes, including *RUNX*2, *OSX*, and *OCN* after 14 days of treatment, with decreased expression observed after 21 days. Similarly, Curtis et al. have reported that *OCN* expression increases significantly in human MSCs treated with 10 nM vitamin D3 mainly on day 14 [29]. Conversely, Posa et al. have demonstrated that *RUNX*2 expression increases significantly at 7 and 14 days, decreasing after 21 days of vitamin D treatment in stem cells derived from the dental bud (DBSCs) [30]. Our results differ from those obtained by Kim et al., who have observed early changes in *ALP*, collagen-1 (Col-1), and *OCN* expression in MC3T3-E1 cells treated with 1 × 10^−10^, 1 × 10^−12^, and 1 × 10^−14^ M of 1,25-dihydroxyvitamin D3 [21]. These differences could be attributed to the dose range and the type of cells analyzed. Furthermore, Wan Hasan et al. have observed that genes such as OSX, COL1 alpha-1 (COL1*α*1), *ALP*, and *OCN* significantly increase in cells treated with vitamin D from 3 to 15 days [4].

Regarding vitamin E, few studies have evaluated the expression of different genes involved in osteogenic differentiation, owing to a poor understanding of its mechanism of action and the lack of consensus regarding the appropriate dose for therapeutic effects. In the present study, we observed that vitamin E increased the expression of the *OSX* and *RUNX*2 genes at 14 days, with a decrease observed following 21 days of treatment. Meanwhile, *OCN* increased after 14 days and remained elevated even after 21 days of treatment. In a study performed by Ahn et al., 68 genes involved in osteogenesis and osteoclastogenesis were evaluated during differentiation of MSCs treated with vitamin E, and *RUNX*2 demonstrated a 1.5-fold increase in expression following vitamin E treatment, suggesting that vitamin E had a positive effect on the expression of osteogenic differentiation genes in MSCs [24]. Conversely, Wan Hasan et al. have determined that treatment with annatto-derived vitamin E induces an increase in the expression of osteoblastic differentiation genes such as *OSX*, *COL*1*α*1, *ALP*, and *OCN*, depending on the time of treatment [4].

In the present study, the vitamins D and E combination induced a decrease in cell proliferation (49%) greater than that observed with individual treatments, with evident morphological changes, demonstrating larger cells and with extensive prolongations. Additionally, we observed a minimal increase in the formation of calcified nodules when the two vitamins were applied concurrently, which was associated with an increase in the expression of osteoblastogenesis marker genes, including *RUNX*2, *OSX*, and *OCN*, which may suggest a synergistic and nonadditive effect on the induction of osteoblastic differentiation in MSCs.

There are no reports in the literature on the effect of concurrent applications of vitamins D and E on mesenchymal cells. To date, vitamins A and D have been compared in a study by Bosetti et al., reporting that osteoblast-like cells can respond to differentiation-inducing stimuli at any stage of their lifetime; however, they failed to evaluate the concurrent use of both vitamins [31]. Additionally, Gigante et al. have observed that vitamin K alone or in combination with vitamin D induces cell differentiation in MSCs. The combination of both vitamins generates a synergistic effect in the differentiating these stem cells toward osteoblasts [32].

## 5. Conclusions

Our findings suggest that vitamins D and E induce the osteoblastic differentiation of hDPSCs, as demonstrated by decreased cell proliferation, morphological changes, and the formation of calcified nodules, with an increased expression of differentiation genes such as *RUNX*2, *OSX*, and *OCN*. Further, simultaneous treatment with vitamins D + E induces a synergistic effect in differentiation toward an osteoblastic lineage.

## Figures and Tables

**Table 1:** Primers used in this study.

Name	Forward Reverse
RUNX2	5′-CATCTAATGACACCACCAGGC-3′
	5′-GCCTACAAAGGTGGGTTTGA-3′
OSX	5′-TGGGAAAAGGGAGGGTAATC-3′
	5′-CGGGACTCAACAACTCTGG-3′
OCN	5′- CCTCACACTCCTCGCCCTAT-3′
	5′- TCCCAGCCATTGATACAGGT-3′
GAPDH	5′- GAAGGTGAAGGTCGGAGTC-3
	5′-GAAGATGGTGATGGGATTTC-3

RUNX2, runt-related transcription factor 2; OSX, Osterix; OCN, Osteocalcin; GAPDH, glyceraldehyde 3-phosphate dehydrogenase.

## Data Availability

The data used to support the findings of this study are available from the corresponding author upon request.
